# Hearing in catfishes: 200 years of research

**DOI:** 10.1111/faf.12751

**Published:** 2023-04-20

**Authors:** Friedrich Ladich

**Affiliations:** ^1^ Department of Behavioral and Cognitive Biology University of Vienna Vienna Austria

**Keywords:** audiogram, communication, eardrum, inner ear, noise, Weberian ossicles

## Abstract

Ernst Weber stated in 1819, based on dissections, that the swimbladder in the European wels (*Silurus glanis*, Siluridae) and related cyprinids serves as an eardrum and that the ossicles connecting it to the inner ear function as hearing ossicles similar to mammals. In the early 20th century, K. von Frisch showed experimentally that catfishes and cyprinids (otophysines) indeed hear excellently compared to fish taxa lacking auxiliary hearing structures (ossicles, eardrums). Knowledge on hearing in catfishes progressed in particular in the 21st century. Currently, hearing abilities (audiograms) are known in 28 species out of 13 families. Recent ontogenetic and comparative studies revealed that the ability to detect sounds of low‐level and high frequencies (4–6 kHz) depends on the development of Weberian ossicles. Species with a higher number of ossicles and larger bladders hear better at higher frequencies (>1 kHz). Hearing sensitivities are furthermore affected by ecological factors. Rising temperatures increase, whereas various noise regimes decrease hearing. Exposure to high‐noise levels (>150 dB) for hours result in temporary thresholds shifts (TTS) and recovery of hearing after several days. Low‐noise levels reduce hearing abilities due to masking without a TTS. Furthermore, auditory evoked potential (AEP) experiments reveal that the temporal patterns of fish‐produced pulsed stridulation and drumming sounds are represented in their auditory pathways, indicating that catfishes are able to extract important information for acoustic communication. Further research should concentrate on inner ears to determine whether the diversity in swimbladders and ossicles is paralleled in the inner ear fine structure.

**Table:** 

1. INTRODUCTION	618
2. ANATOMY AND FUNCTION OF THE INNER EAR	621
2.1. Ear anatomy	621
2.2. Sound detection	621
3. EFFECTS OF SWIMBLADDERS AND WEBERIAN OSSICLES ON HEARING	623
3.1. Extirpation and mutilation	623
3.2. Ontogeny	624
3.3. Diversity in swimbladders and Weberian ossicles	625
4. ECOLOGICAL CONSTRAINTS ON HEARING	625
4.1. Temperature	625
4.2. Noise exposure	627
4.3. Noise masking	628
5. DETECTION OF CONSPECIFIC SOUNDS	629
5.1. Neural representation of sounds within auditory pathways	629
5.2. Sex‐specific differences in sounds detection	629
6. MISCELLANEOUS	630
6.1. Functional significance of hearing in catfishes	630
6.2. Echolocation	630
6.3. Albinism	630
7. SUMMARY AND FUTURE RESEARCH	631
ACKNOWLEDGMENTS	631
DATA AVAILABILITY STATEMENT	631
REFERENCES	632

## 1 INTRODUCTION

Catfishes belong, together with cypriniforms, characiforms and gymnotiforms, to the otophysines. This successful group of bony fishes comprises more than 10,000 species (in comparison: mammals fewer than 6000 species) that are primarily characterized by anatomical structures for improvement of their hearing. Very similar to four‐limbed vertebrates (tetrapods), otophysines possess hearing ossicles connected to the swimbladder, which serves (in addition to its role in buoyancy) as an ear drum (tympanum). Tympana enable vertebrates (and insects) to detect sound pressure changes in a sound field. Sound pressure changes result in volume changes of gas‐filled cavities such as swimbladders, which are paralleled by oscillations of the walls of these bladders. Such oscillations are directly picked up by a series of tiny ossicles and transmitted to the inner ear fluids (perilymph and endolymph). Thus, the anterior walls of swimbladders function as an eardrum when oscillations are transmitted to the inner ears via auditory ossicles. In addition, fish are acoustically transparent and move back and forth in parallel with water particles in a sound field. The dense otolith lags behind, creating a bending of ciliary bundles in sensory maculae and thus the detection of particle motion in parallel to sound pressure. Two‐hundred years ago, Weber (1819, 1820) assumed, based on anatomical studies, that the anterior swimbladder wall functions as an ear drum and that ossicles connecting it to the inner ear serve as hearing ossicles, similar to mammals. It took another 100 years and numerous fierce disputes before Weber was proved right. Von Frisch and his collaborators showed that brown bullhead catfish (*Ameiurus nebulosus*, Ictaluridae) and the Eurasian minnow (*Phoxinus phoxinus*, Leuciscidae) are more sensitive than pike, perch, trout and eels, whose swimbladders are not connected to the inner ear. Subsequently, the auditory ossicles of otophysines were named Weberian ossicles in honour of Weber; together with ligaments and the swimbladder, they constitute the Weberian apparatus. Note that otophysic connections, namely connections between ears and swimbladders, are not limited to fishes possessing a Weberian apparatus. Functional coupling between the swimbladder (or other gas‐filled cavities) and the inner ear are also known in some cichlids, holocentrids, sciaenids, herrings, mormyrids, pempherids and labyrinth fishes. This involves anterior swimbladder extensions or cavities directly attached to the inner ear (Braun & Grande, 2008; Ladich, 2016; Ladich & Schulz‐Mirbach, 2016; Popper & Fay, 1999; Radford et al., 2013).

The number of studies on hearing in catfishes increased considerably over the last 25 years. By the late 1990s, baseline hearing abilities were known in only two catfish species, the hardhead sea catfish (*Ariopsis felis*, Ariidae) and the brown bullhead from two families (Fay, 1988). Currently, baseline hearing abilities have been measured in 28 catfish species out of 13 families (Table 1). This increase in data reflects the introduction of a new method to measure hearing thresholds throughout the hearing range (audiograms) in fishes, the electrophysiological auditory evoked potential (AEP) recording technique by Kenyon et al. (1998). Corwin et al. (1982) showed that auditory brainstem responses (ABRs) are present in five vertebrate classes (elasmobranchs, osteichthyans, amphibians, reptiles and birds) by recording potentials generated in the auditory pathway in the presence of sound. Despite Corwin et al.'s (1982) observation, hearing thresholds and entire audiograms had, up to end of the 20th century, been determined using behavioural techniques. These require training fish to respond to sound stimuli either by swimming across a barrier, pecking a pedal to get a food reward, or measuring changes in heart or breathing rates (Corwin, 1981; Fay, 1969; Tavolga & Wodinsky, 1963; Yan & Popper, 1991). In contrast to behavioural techniques, the AEP technique measures the response of the auditory pathway from the inner ear up to the midbrain or forebrain. Different techniques applied resulted in differences between audiograms. According to Ladich and Fay (2013), there is no “factor” for estimating behavioural thresholds from AEP measurements. Note here that differences exist between behavioural audiograms and between AEP audiograms depending on the techniques applied (see section 3.1: Extirpation and mutilation). So far, audiograms have not been measured in any catfish species using both behavioural and AEP techniques.

**TABLE 1:** Systematic overview of catfish species whose baseline hearing sensitivities have been measured using various techniques.

Family	Species	Research topics	References
Callichthyidae	Peppered corydoras (*Corydoras paleatus*)	Stridulation sounds, sound spectra	Ladich (1999)
	*False network catfish* (*Corydoras sodalis*)	Weberian ossicles, swimbladder	Lechner and Ladich (2008)
	*Corydoras aeneus* (Bronze corydoras)	Albinism	Lechner and Ladich (2011)
	*Flagtail catfish* (*Dianema urostriatum*)	Weberian ossicles, swimbladder	Lechner and Ladich (2008)
	*Megalechis thoracata*	Both sexes, stridulation sounds, sound spectra	Hadjiaghai and Ladich (2015)
Loricariidae	*Ancistrus ranunculus*	Weberian ossicles, swimbladder	Lechner and Ladich (2008)
	*Hemiodontichthys acipenserinus*	Weberian ossicles, swimbladder	Lechner and Ladich (2008)
	*Hypoptopoma thoracatum*	Weberian ossicles, swimbladder	Lechner and Ladich (2008)
Siluridae	Wels catfish (*Silurus glanis*)	Albinism	Lechner and Ladich (2011)
		Temperature	Maiditsch and Ladich (2014)
Malapteruridae	*Malapterurus beninensi*	Weberian ossicles, swimbladder	Lechner and Ladich (2008)
Mochokidae	*Synodontis schoutedeni*	Weberian ossicles, swimbladder	Lechner and Ladich (2008)
		Ontogeny, stridulation sounds, sound spectra	Lechner et al. (2010)
Claroteidae	African bullhead (*Lophiobagrus cyclurus*)	Ontogeny, Weberian ossicles	Lechner et al. (2011)
Ariidae	*Tete sea catfish* (*Ariopsis seemani*)	Weberian ossicles, swimbladder	Lechner and Ladich (2008)
	Hardhead sea catfish (*Ariopsis felis*)*	Inner ear anatomy	Popper and Tavolga (1981)
Doradidae	Southern striped Raphael (*Platydoras armatulus*)	Drumming and stridulation sounds, sound spectra	Ladich (1999)
		White noise masking	Wysocki and Ladich (2005)
		Temperature, stridulation sounds	Papes and Ladich (2011)
	Whitebarred catfish (*Agamyxis pectinifrons*)	Weberian ossicles, swimbladder	Lechner and Ladich (2008)
		Drumming and stridulation sounds, sound spectra	Ladich (1999)
		Swimbladder	Zebedin and Ladich (2013)
	Talking catfish (*Acanthodoras spinosissimus*)	Swimbladder	Zebedin and Ladich (2013)
	*Amblydoras affinis*	Swimbladder	Zebedin and Ladich (2013)
	*Hemidoras morrisi*	Swimbladder	Zebedin and Ladich (2013)
	*Megalodoras uranoscopus*	Swimbladder	Zebedin and Ladich (2013)
		Temperature, acclimation	Schliwa and Ladich (2021)
	Ripsaw catfish (*Oxydoras niger*)	Swimbladder	Zebedin and Ladich (2013)
Auchenipteridae	Striped woodcat (*Trachelyopterichthys taeniatus*)	Weberian ossicles, swimbladder	Lechner and Ladich (2008)
Ictaluridae	Channel catfish (*Ictalurus punctatus*)	Temperature	Wysocki et al. (2009)
		Exposure to mid‐frequency naval sonar	Halvorsen et al. (2012)
	Brown bullhead (*Ameiurus nebulosus*)*	Extirpation of ossicles	Poggendorf (1952)
		Swimbladder deflation	Weiss et al. (1969)
Heptapteridae	*Pimelodella* sp.	Weberian ossicles, swimbladder	Lechner and Ladich (2008)
Pimelodidae	Bloch's catfish (*Pimelodus blochii*)	Drumming and stridulation sounds, sound spectra	Ladich (1999)
	Pictus cat (*Pimelodus pictus*)	Drumming and stridulation sounds, sound spectra	Ladich (1999)
		White noise exposure, TTS	Amoser and Ladich (2003)
		Temperature, acclimation	Wysocki et al. (2009)
Pseudopimelodidae	*Batrochoglanis raninus*	Weberian ossicles, swimbladder	Lechner and Ladich (2008)

*Note*: Asterisks indicate that behavioural techniques have been applied. In other studies AEP recording techniques were used. Common names (if available) and scientific names according to fishbase.de. Systematics followed that of Nelson et al. (2016). The Research topics column describes topics of the study beyond determining baseline hearing thresholds. For more information, see original articles.

Besides measuring hearing abilities (thresholds) under quiet laboratory conditions, behavioural and the AEP recording technique enable researchers to study numerous additional questions on hearing in catfishes. First, these approaches helped to answer the question of how the Weberian apparatus affects hearing in catfishes. The AEP technique was used to analyze how the ontogenetic development and diversity of the Weberian ossicles (Chardon, 1968) and swimbladders affect hearing sensitivity. Furthermore, the effects of ecological constraints, in particular ambient temperature and aquatic noise, on hearing were studied in several catfish families. Finally, the comparison between hearing curves and sound spectra and the analysis of AEPs in response to conspecific sounds enables assessing if and to what degree temporal patterns of stridulation and drumming sounds are represented in the auditory pathways and can be used for acoustic communication in catfishes. These and additional topics including sex‐specific differences, echolocation and albinism are dealt with in the following.

## 2 ANATOMY AND FUNCTION OF THE INNER EAR

### 2.1 Ear anatomy

The ear in teleost fishes is composed of a dorsal part (pars superior) including the utricle and three semicircular canals and the ventral part (pars inferior) consisting of the saccule and the lagena. The utricle, saccule and lagena are often termed otolithic endorgans because each consists of an otolith, which is a calcareous structure that overlies a sensory epithelium (macula). The saccule and its otolith (sagitta) are typically ovoid and the largest otolithic endorgan, whereas the lagena and its otolith (asteriscus) is the smallest; the utricle and its otolith (lapillus) are small to intermediate‐sized. Maculae are oriented horizontally in the utricle and vertically in the saccule and lagena. Maculae consist of different types of hair cells that are oriented in different patterns within maculae (Lanford et al., 2000; Popper & Fay, 1999; Schulz‐Mirbach et al., 2019; Schulz‐Mirbach & Ladich, 2016).

**FIGURE 1:**
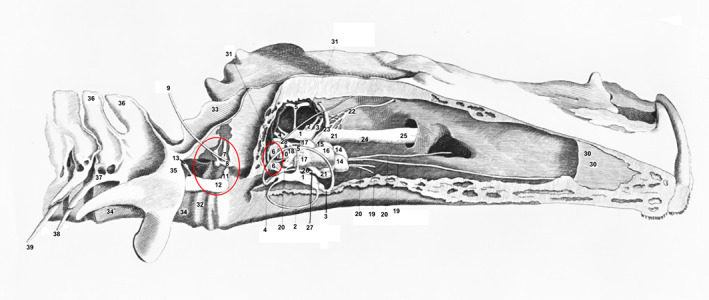
Medial dissection of the head of the Wels catfish showing the Weberian ossicles, auditory apparatus and brain. 1 – utriculus; 2 – horizontal canal; 3 – anterior canal; 4 – posterior canal, 5 – crus commune, 6 – sacculus, 7 – locus sinus impar, 8 – scaphium (stapes Weberi), 9 – probe entering sinus imparis, 10 – probe leaving sinus imparis, 11 – intercalarium (incus Weberi), 12 – anterior end of tripus (malleus Weberi), 13 – medial (vertebral) process of tripus, 14 – olfactory lobes, 15 – optic lobe, 16 – anterior cerebellum, 17 – lateral cerebellum, 18 – medulla, 19 – olfactory tract, 20 – optic nerve, 21, 22, 23 – trigeminal nerve branches, 24 – locus of intraorbital trunk, 25 – trigeminal ophthalmic tract, 26 – auditory nerve, 27 – nerve ramus to trigeminal, 28 – vagus nerve, 30 – nasal septum, 31 – bony labyrinth, 32 – first vertebrum, 33 – neural spine, 34 – fused vertebra 2 and 3, 35 – transverse process of 2nd vertebrum, 36 – fused neural spines, 37 – transverse process of 3rd vertebrum, 38, 39 – ribs. Adapted from Weber (1820). Note, that the swimbladder and the connection between the Weberian ossicles (red circles) and the inner ears are not shown. The large red circle indicates the Weberian ossciles on the right side and the small circle the sacculi of both inner ears. These otolithic endorgans are connected via the sinus imparis to the Weberian ossicles.

**FIGURE 2:**
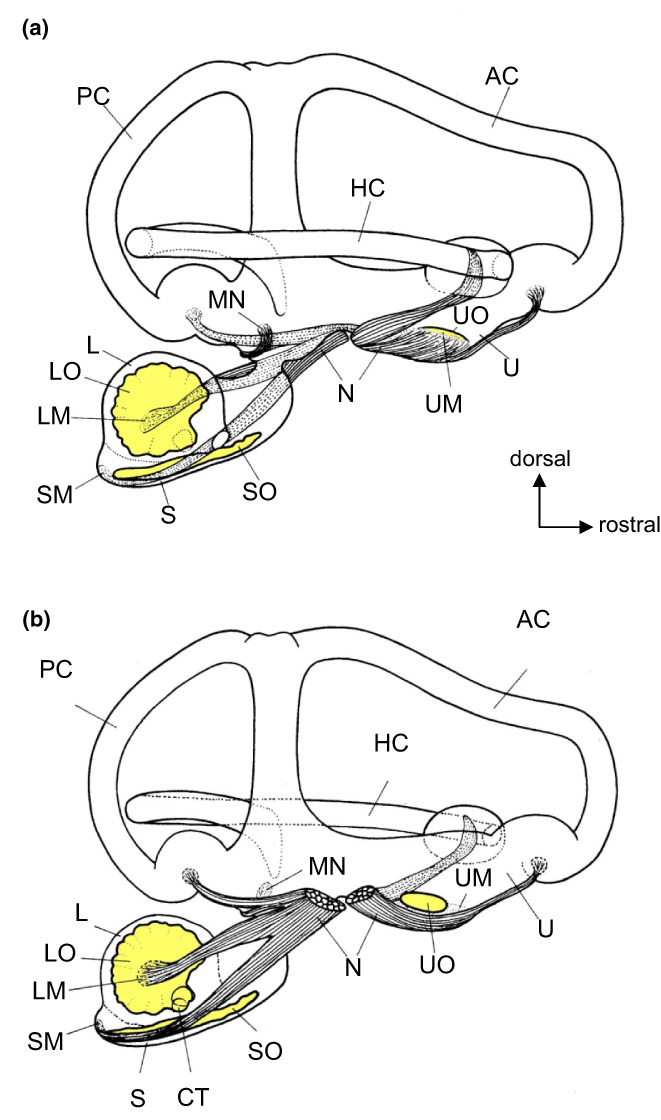
Lateral (a) and medial (b) view of the right ear of the Wels catfish. Anterior is to the right. AC, anterior semicircular canal; CT, canalis transversus; HC, horizontal semicircular canal; L, lagena; LM, lagenar macula; LO, lagenar otolith; MN, macula neglecta; N, vestibular and auditory branches of the eighth nerve; PC, posterior semicircular canal; S, sacculus; SM, saccular macula; SO, saccular otolith; U, utriculus; UM, utricular macula; UO, utricular otolith. Redrawn after Retzius (1881) and modified after Ladich and Popper (2004).

The first detailed anatomical studies on catfish ears were conducted by Weber (1820) (Figure 1) and Retzius (1881) (Figure 2), both in the European Wels catfish (*Silurus glanis*, Siluridae). These and other studies in otophysines revealed that otolithic endorgans differ considerably from the majority of teleosts. In otophysines, the lagena is the largest endorgan, while the saccule is a lengthy compartment containing a stick‐like sagitta with extensions (flutes) (Figure 2). Interestingly, the morphology of the inner ear of one species, the hardhead sea catfish, differs considerably from the general catfish and otophysine pattern. In that species, the utricle is very large and its sensory epithelium consists of a band around the equatorial region of the otolithic chamber (Popper & Tavolga, 1981). These structural modifications are potentially linked to the hearing in marine catfishes (Popper & Tavolga, 1981).

### 2.2 Sound detection

**FIGURE 3:**
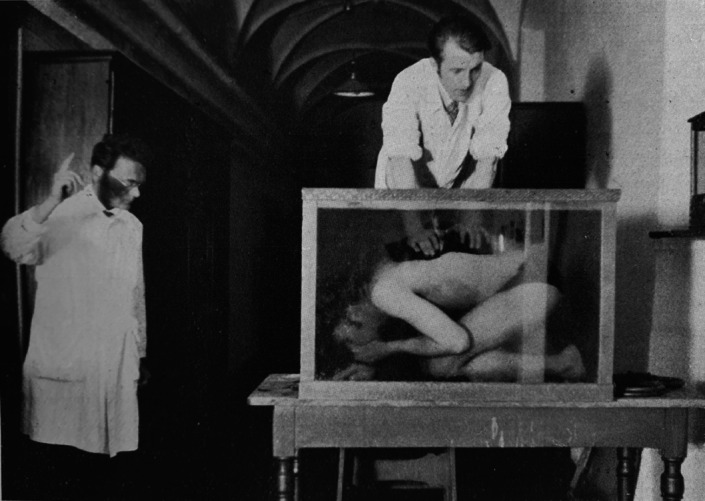
Experimental setup demonstrating that catfish (and minnows) hear better than humans under similar conditions. Brown bullheads detected whistling sounds at distances of up to 65 m (minnows up to 75 m) as compared to 50 m in humans. The future Nobel laureate Karl von Frisch is standing on the left. Experiments were conducted in a 120‐m‐long hallway at the Institute of Zoology at the University of Munich. From Stetter (1929).

While the basic anatomy of the catfish and other fish inner ears were known at the end of the 19th century (Retzius, 1881), the question remained whether fish are able to hear sound. Fish reacted or did not react to loud artificial sounds generated by bells or whistles in the air or to plucking strings attached to aquaria (Parker, 1918). Ultimately, a catfish helped to answer the question unequivocally. A brown bullhead named Xaverl was regularly fed while the future Nobel laureate Karl von Frisch was whistling. After some time, the catfish swam to the water surface as soon it heard a whistle – without any further stimulus (Von Frisch, 1923). Using this food reward conditioning, numerous species from various taxa including minnows, pikes, perches, trout, eels and others consistently responded to artificial sounds, proving that fish have a well‐developed sense of hearing (Von Frisch, 1936, 1938). Von Frisch and his collaborators furthermore demonstrated that otophysines hear much better than other taxa – even better than humans under similar conditions. Brown bullheads and Eurasian minnows in aquaria responded at greater distances to whistles than humans in aquaria (Figure 3) (Stetter, 1929). Both catfish and minnows could be trained to discriminate between pure tones of different frequencies (Tavolga, 1982). In addition, Von Frisch showed that removing the swimbladder in minnows reduced the “acuteness of the sense of hearing very much” (Von Frisch, 1938).

How do fish detect sound? Theoretical considerations assume that the entire fish vibrates in a sound field because its density is very similar to that of the surrounding water (Hawkins, 1993; Hawkins & Myrberg, 1983; Kalmijn, 1988). Only otoliths lag behind due to their threefold higher density. The relative motion between sensory epithelia and otoliths bends the ciliary bundles of hair cells and subsequently stimulates hair cells. This process enables all taxa to detect the particle motion in a sound field at high sound levels up to 1000 hertz. Detecting the pressure fluctuations in a sound field by connecting the inner ear to oscillating walls of gas‐filled cavities potentially increases the motion of inner ear structures, thus widening the hearing abilities considerably. Fish lacking swimbladders such as flatfishes are unable to detect sound pressure and are limited to particle motion hearing (Hawkins, 1993). Fishes possessing auxiliary (accessory) hearing structures are usually called hearing specialists (Popper et al., 2022). Besides otophysines, hearing specialists comprise all taxa having a connection between a gas‐filled cavity (e.g. swimbladder) and the inner ear. Some holocentrids and cichlids possess an anterior extension of the swimbladder, which increases their sensitivity (Ladich & Schulz‐Mirbach, 2016). Interestingly, even taxa without a clear connection between the swimbladder and the inner ear such as damselfish (family Pomacentridae) can be sound pressure sensitive (Myrberg & Spires, 1980).

**FIGURE 4:**
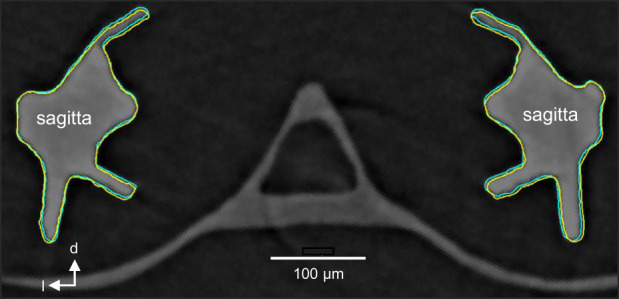
Movement of auditory structures in a sound field. Photograph of transverse section show movement of saccular otoliths in glass catfish *Kryptopterus vitreolus* when pure tones of 350 Hz were played back. The overlay of outer contours of otoliths reveals slight rotational movement of sagittae. Modified after Maiditsch et al. (2022). See also film clip in supplements of this paper.

The anatomy of the auxiliary hearing structures in otophysines constitutes the most complex structure for hearing enhancement among fishes. Oscillations of the anterior swimbladder walls are transmitted via 1–4 bony (Weberian) ossicles (and their ligaments) to the perilymphatic fluid of the sinus impar and then to the endolymphatic fluid in the transverse canal connecting both saccules (Von Frisch, 1938). While the transmission of vibrations has mainly been a working hypothesis, the recent development of new techniques (X‐ray phase contrast imaging) helped visualize movement of auditory structures (swimbladder wall, ossicles and otoliths) in a sound field. Standing wave, tube‐like setups enable maximizing sound pressure (in the center of the tanks) when both speakers (shakers) are driven in phase, and they maximize particle motion when both speakers are driven out of phase (180°). This approach helped separate sound pressure and sound‐induced particle motion from each other in a sound field experimentally. Subsequently, it is possible to determine which sound component affects the motion of auditory structures. Schulz‐Mirbach et al. (2020) showed in the goldfish (*Carassius auratus*, Cyprinidae) that all auditory structures move more when sound pressure is maximized versus maximization of particle motion. The endolymphatic fluid flow from the transverse canal into the saccules resulted in a tilting and dorsoventral motion of the sagittae, which most likely stimulates sensory hair cells due to the vertically oriented ciliary bundles. Synchrotron radiation‐based tomography with high spatio‐temporal resolution showed in the glass catfish *Kryptopterus vitreolus* (Siluridae) a rotational movement of the sagittae, similar to goldfish (Maiditsch et al., 2022) (Figure 4). This supports the hypothesis that the otophysan saccule is an endorgan specialized to detect sound pressure, whereas the lagena and utricle detect particle motion.

The detectable frequency range of catfishes is wider than in most fish species except in Alosinae, a subfamily of clupeiforms (herrings, shads), which detect ultrasound up to 180 kHz (Mann et al., 2001). Currently, no empirical study has measured the upper frequency limit in catfishes. Partly contradictory observations have been reported in brown bullheads: Stetter (1929) observed that three specimens of brown bullhead responded up to 13 kHz. In contrast, Eurasian minnows, which also belong similarly to otophysines, had an upper frequency limit between 4645 and 6960 Hz. Poggendorf (1952) reported a large variation between specimens, with just one out of 10 brown bullheads hearing frequencies up to 10,000 Hz. Weiss et al. (1969) observed that all brown bullhead hear from 100–4000 Hz. These differences between studies may reflect different techniques applied. Nevertheless, an upper frequency limit of at least 5000 Hz was reported in almost all 28 catfish species studied so far (see review by Ladich & Fay, 2013).

## 3 EFFECTS OF SWIMBLADDERS AND WEBERIAN OSSICLES ON HEARING

### 3.1 Extirpation and mutilation

Weber (1820) named the ossicles connecting the swimbladder to the inner ear in fishes similar to those in mammals, namely malleus, incus and stapes, to indicate their auditory function (Figure 1). Because auditory ossicles in fishes are not derived from ancient jaw bones but from anterior vertebrae, they were renamed to tripus, intercalarium and scaphium by Bridge and Haddon (1889). It took 100 years before Weber's hypothesis was confirmed by showing that removing swimbladders resulted in a hearing loss (Stetter, 1929; Von Frisch, 1936, 1938).

In order to analyze the function of the Weberian apparatus in more detail, hearing sensitivity must be measured at several frequencies across the hearing range at quiet conditions using standardized units. Today, hearing sensitivity or hearing thresholds are typically measured as sound pressure levels in dB re 1 μPa. Prior studies used other units (e.g. Fay, 1988: dB re 1 dyne/cm^2^), which can be converted accordingly (Ladich & Fay, 2013).

**FIGURE 5:**
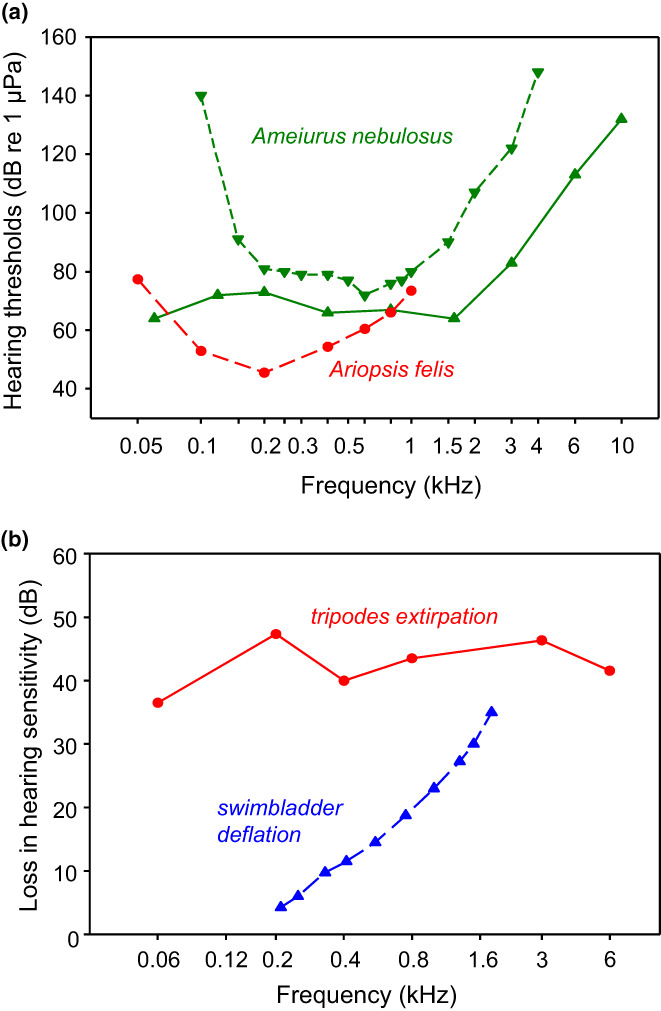
Studies using behavioural techniques to investigate hearing in catfishes. (a) Baseline audiograms of the brown bullhead and the hardhead sea catfish by different authors: triangles up, brown bullhead by Poggendorf (1952) and triangles down by Weiss et al. (1969), and hardhead sea catfish by Popper and Tavolga (1981). (b) Experiments studying the hearing loss after eliminating parts of the Weberian apparatus in the brown bullhead. Extirpation of both tripodes resulted in a frequency‐independent hearing loss. In contrast, hearing loss increased with frequency after swimbladder mutilation. Calculated after Poggendorf (1952) and Kleerekoper and Roggenkamp (1959).

No standard way to measure hearing abilities in fish or other animals exists, in contrast to humans for whom physicians use audiometers. Thus, investigators developed various techniques to determine hearing thresholds. Audiograms based on behavioural techniques were determined only in two out of several thousand catfish species until the late 1980s, namely in the brown bullhead and the hardhead sea catfish (Fay, 1988; Ladich & Fay, 2013). Baseline hearing thresholds measured by Poggendorf (1952) and Weiss et al. (1969) in the brown bullhead differed somewhat in the frequency range measured as well as in absolute hearing thresholds (by up to 40 dB) at different frequencies, most likely reflecting different techniques. Poggendorf (1952) used a loudspeaker pointing upwards at the tank bottom and food reward conditioning. In contrast, Weiss et al. (1969) used two speakers facing each other and electric shock‐avoidance training (Figure 5a). Despite the differences between the audiograms of the brown bullhead, the assumption is that its hearing abilities differ from that of the hardhead sea catfish. The brown bullhead seems to be most sensitive above 500 Hz, whereas the hardhead sea catfish had its lowest thresholds at 200 Hz. This might be due to differences in the anatomy of their inner ears (see above: Popper & Tavolga, 1981).

Moreover, different methods may explain why the degree of hearing improvement by the Weberian apparatus in the brown bullhead differs between studies. Poggendorf (1952) removed both tripodes, the most caudal and largest Weberian ossicles (Figure 1) and left the swimbladder intact. The hearing sensitivity dropped by 36–47 dB without a clear relationship between frequency and hearing loss (Figure 5b). In contrast, Kleerekoper and Roggenkamp (1959) deflated the swimbladder by cutting out a piece of its wall and observed a sensitivity loss that was highly frequency dependent (Figure 5b). The hearing sensitivity decreased by 4 dB at 210 Hz up to 35 dB at 1840 Hz. These differences in hearing loss raise the question of whether the hearing enhancement by the Weberian ossicles depends on the frequency or on the accessory auditory structures that were eliminated or on the technique chosen. A third elimination study was on the channel catfish (*Ictalurus punctatus*, Ictaluridae) and involved filling the swimbladder with water and measuring potentials in the saccules in response to sounds (Fay & Popper, 1975). Those authors, who did not give thresholds before and after surgery, stated that “there was a considerable loss of sensitivity at all frequencies above 100 Hz with losses generally 30 dB or greater above 200 Hz”.

Elimination studies in the goldfish also failed to answer the question if the Weberian apparatus increases auditory sensitivity with increasing frequency in otophysines. Removal of the entire swimbladder (Fay & Popper, 1974) or of the gas within the swimbladder (Yan et al., 2000) or extirpation of both tripodes (Ladich & Wysocki, 2003) gave contradictory results.

### 3.2 Ontogeny

**FIGURE 6:**
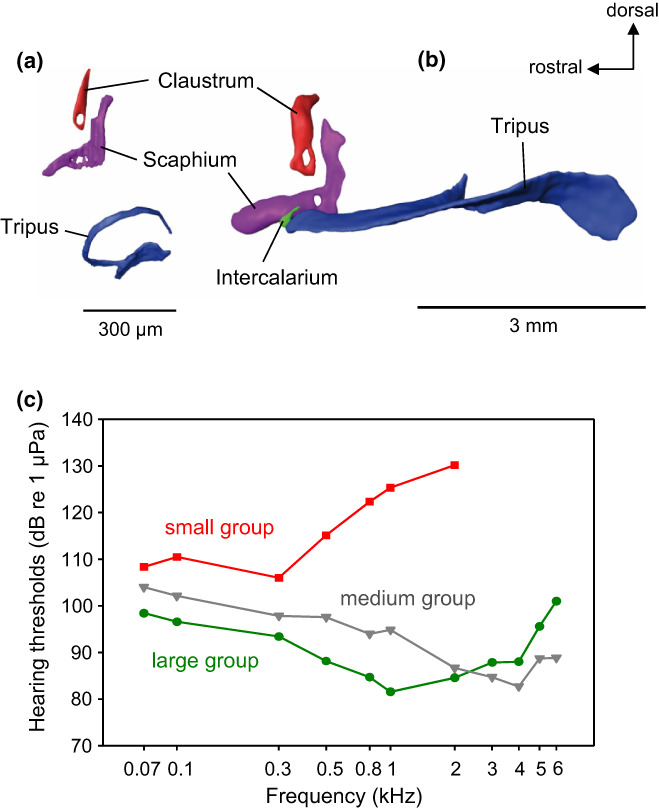
Ontogenetic development of Weberian ossicles and hearing in the African bullhead. (a) Lateral view of isolated Weberian ossicles of a specimen of 11.3 mm standard length (SL) and (b) of an 85.5 mm SL specimen. 3D‐reconstruction based on serial semithin section photomicrographs. Anterior is to the left. (c) Mean hearing thresholds of different size groups. Red: SL = 11.3–15.3 mm, Gray: SL = 39–48 mm, Green: SL = 69–84 mm. Note the increase in auditory sensitivity and in the frequency range in the medium and large groups. Modified from Lechner et al. (2011).

The development of hearing with increasing body size has been measured in two catfish species from two different families. Hearing thresholds were measured in the African bullhead (*Lophiobagrus cyclurus*, Claroteidae) together with the anatomy of the Weberian apparatus in specimens ranging from 11–85.5 mm in standard length. Weberian ossicles and interossicular ligaments were fully developed in all stages except the smallest size group (Figure 6a,b). In the latter, the intercalarium and interossicular ligaments were missing, and the tripus was not fully developed (Figure 6a). Smallest juveniles had lowest hearing sensitivity and were unable to detect frequencies above 2 or 3 kHz. In contrast, thresholds decreased up to 40 dB in the larger individuals, and frequencies were detected up to 6 kHz (Figure 6c) (Lechner et al., 2011). Thus, auditory sensitivity increased significantly as soon as all Weberian ossicles and interossicular ligaments were developed, and the chain of ossicles was complete for transmitting sounds from the swimbladder to the inner ear. In the size groups possessing all Weberian ossicles and capable of perceiving frequencies up to 6 kHz, larger individuals had better hearing abilities at low frequencies (0.05–2 kHz), whereas smaller individuals showed better hearing at the highest frequencies (4–6 kHz).

The second ontogenetic study was conducted on the squeaker catfish *Synodontis schoutedeni* (Mochokidae) in the size group 22–36 mm up to 16–127 mm (standard length). Catfishes of all size groups were able to detect frequencies between 50 Hz and 6 kHz. Best hearing abilities were between 0.3 and 1 kHz except for the smallest group, which heard best at 2 and 3 kHz (Lechner et al., 2010). Body size and hearing sensitivities were significantly correlated at most frequencies. At lower frequencies (0.05 to 2 kHz), large animals showed better hearing, whereas the opposite was the case at 5 and 6 kHz, at which smaller animals had lower hearing thresholds. Comparing audiograms and absolute spectra of stridulation sounds revealed best hearing abilities at frequencies where main energies of stridulation sounds were concentrated: all size groups were able to detect sounds of conspecifics (Lechner et al., 2010). Studies on more catfish species are needed to get a better insight into the development of auditory sensitivities in catfishes and otophysines.

### 3.3 Diversity in swimbladders and Weberian ossicles

**FIGURE 7:**
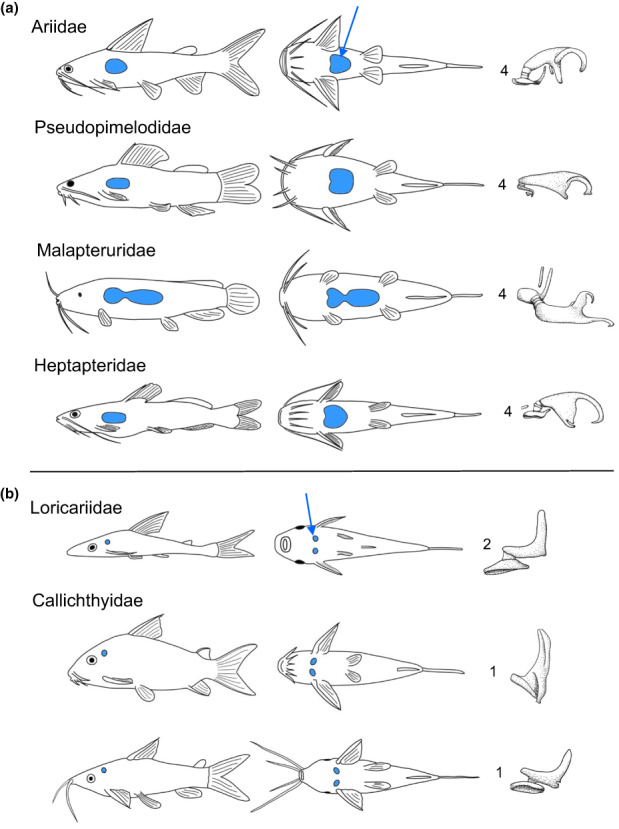
Lateral and ventral view of (a) four species possessing free swimbladders and of (b) three species with paired and encapsulated swimbladders. Weberian ossicles and their number in each species are provided on the right. Swimbladders are in blue and indicated by arrows. (a) Representatives of the families Ariidae (*Ariopsis seemanni*), Pseudopimelodidae (*Batrochoglanis raninus*), Malapteruridae (*Malapterurus beninensis*) and Heptapteridae (*Pimelodella* sp.); (b) Members of the families Loricariidae (*Hypoptopoma thoracatum*) and Callichthyidae (*Corydoras sodalis*, *Dianema urostriatum*). Modified after Lechner and Ladich (2008).

Catfishes exhibit a large variety in swimbladder and Weberian ossicle morphology. Neither organs have been completely lost in any of the more than 10,000 otophysine species. Numerous catfish families have large single, unencapsulated (free) gasbladders and up to four Weberian ossicles. In contrast, many groups have tiny, paired swimbladders located on both sides of the vertebral column, directly behind the cranium, and one or two ossicles (Figure 7). These tiny bladders are surrounded by bony capsules derived from the skull and vertebrae (Alexander, 1964; Bleckmann et al., 1991; Bridge & Haddon, 1889, 1892, 1893; Chardon, 1968; Chardon et al., 2003; Chranilov, 1929). These diverse structures prompted Bridge and Haddon (1889, 1892, 1893) to split catfishes into two groups, the “siluridae normales” with well‐developed gasbladders and the “siluridae abnormales” with reduced bladders. Ladich (1999) observed that members of “normal catfishes” such as pimelodids and doradids hear better than the peppered corydoras (*Corydoras paleatus*, Callichthyidae), which are a member of the “abnormal” callichthyids. In order to determine if these anatomical differences affect hearing in general, Lechner and Ladich (2008) compared 11 representatives from 8 families having well‐developed swimbladders as well as tiny encapsulated ones. Representatives of the families Ariidae, Pseudopimelodidae, Malapteruridae, Heptapteridae, Mochokidae and Auchenipteridae possess large non‐encapsulated bladder (Figure 7a).

**FIGURE 8:**
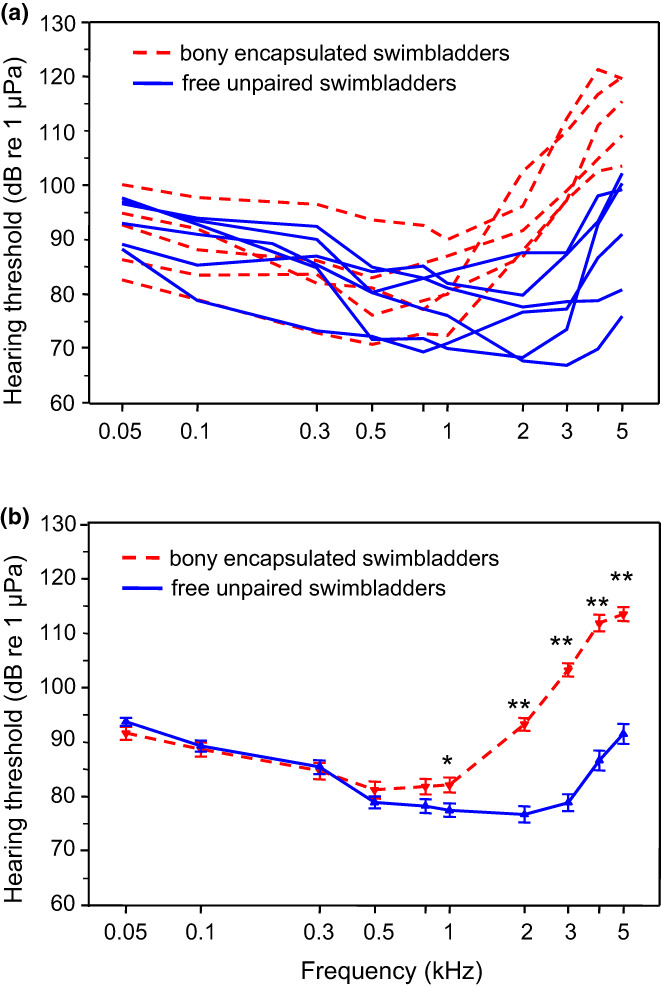
(a) Mean hearing thresholds of six catfish species with free, large unpaired swimbladders (solid lines) and of five species with encapsulated, tiny and paired swimbladders (dashed lines). (b) Mean (± *SE*) hearing thresholds of all species with free swimbladders and paired encapsulated bladders. Asterisks: statistically significant differences between these two groups at particular frequencies. Modified from Lechner and Ladich (2008).

In contrast, members of the families Callichthyidae and Loricariidae have tiny paired and encapsulated bladders with just one or two auditory ossicles and thus a significantly shorter ossicular chain (Figure 7b). Measurements of the auditory sensitivities revealed that all 11 species investigated detect frequencies between 50 Hz and 5 kHz (Figure 8a). The lowest absolute sensitivity was found in the ariid catfish (67 dB re 1 μPa) and the highest in a callichthyid catfish (121 dB). The mean auditory thresholds of all six species having large bladders and all 5 species having tiny bladders revealed that “normal” catfish hear significantly better than “abnormal” ones between 1 and 5 kHz, but not at lower frequencies (Figure 8b). Furthermore, species with larger swimbladders had lower thresholds (thus were more sensitive) than others, and a longer ossicular chain and more ossicles improved hearing between 3 and 5 kHz (Lechner & Ladich, 2008). These results indicate that swimbladder size and ossicle number improve hearing ability at higher frequencies in catfishes.

Thorny catfishes (family Doradidae) exhibit a large variation in swimbladder morphology. All species possess large unpaired bladders according to the classification given in Figure 7a but nevertheless vary in size, form and may have simple or branched diverticula (Zebedin & Ladich, 2013). The bladder morphology was quantified by measuring its length, width and height and calculating a relative swimbladder length, which differed significantly between species. All species detected frequencies between 70 and 6 kHz. Mean hearing thresholds of species possessing relatively smaller bladders were slightly lower at higher and lower frequencies than those of species having relatively larger bladders. This is partly in contrast to the study by Lechner and Ladich (2008), in which species with large unpaired bladders possessed higher sensitivities at higher frequencies than species having tiny paired and encapsulated bladders (Zebedin & Ladich, 2013).

## 4 ECOLOGICAL CONSTRAINTS ON HEARING

Ecological constraints – in particular temperature and noise (natural ambient or anthropogenic noise) – affect hearing and subsequently limit sound detection and communication in animals including fishes (Ladich, 2018, 2019).

### 4.1 Temperature

**FIGURE 9:**
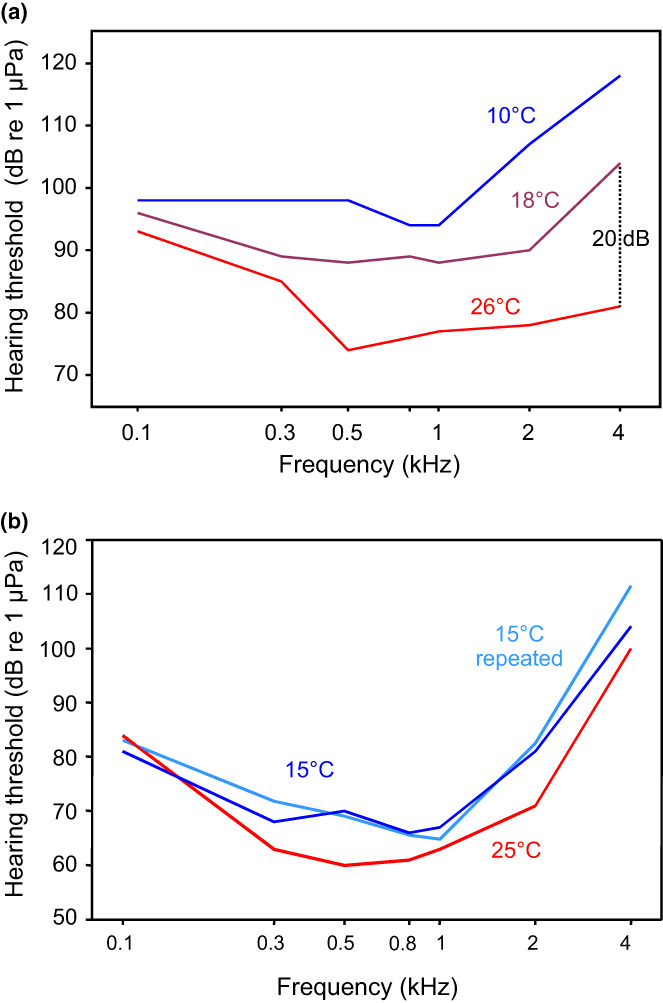
Mean auditory thresholds of eurythermal catfishes after acclimation to different temperatures. (a) Channel catfish after acclimation to 10, 18 and 26°C. Modified after Wysocki et al. (2009). (b) Wels catfish after acclimation to 15, 25 and again 15°C (light blue line). Modified after Maiditsch and Ladich (2014).

Fishes are ectothermic and their body temperature generally depends on ambient water temperature. That temperature variously affects physiological processes and sensory systems. The values in the different environments that fishes inhabit can undergo either considerable or small diurnal and seasonal fluctuations. Stenothermal species tolerate small temperature variations, e.g., tropical species, whereas eurythermal species living in temperate climates have a broader temperatures tolerance. Steno‐ and eurythermal catfishes have been studied in terms of baseline hearing sensitivities, in terms of thermal acclimation, and in terms of temporal resolution of the auditory system. Among eurythermal catfishes, the channel catfish and the Wels catfish were chosen, among stenothermal (tropical) species the Pictus cat (*Pimelodus pictus*, Pimelodidae) and two doradids, namely the Southern striped Raphael (*Platydoras armatulus*, Doradidae) and *Megalodoras uranoscopus* (Doradidae). In general, hearing sensitivity increased with temperature in all species investigated including catfishes (Maiditsch & Ladich, 2014; Papes & Ladich, 2011; Schliwa & Ladich, 2021; Wysocki et al., 2009). However, the extent of threshold shift varied considerably between eurythermal species. While the values decreased maximally by 1 dB at 4 kHz when temperature increased by 1°C in the European wels, the shift was more than 2 dB per 1°C in the channel catfish in a similar temperature range after similar acclimation times (Figure 9a,b) (Ladich, 2018). A similar variation in threshold changes per degree Celsius was observed in the three stenothermal species investigated so far. Comparison of all seven species tested so far (including carp and goldfish) revealed that the shift in auditory sensitivity due to temperature change did not differ significantly between species adapted to different temperature regimes (see figure 13 in Schliwa & Ladich, 2021).

**FIGURE 10:**
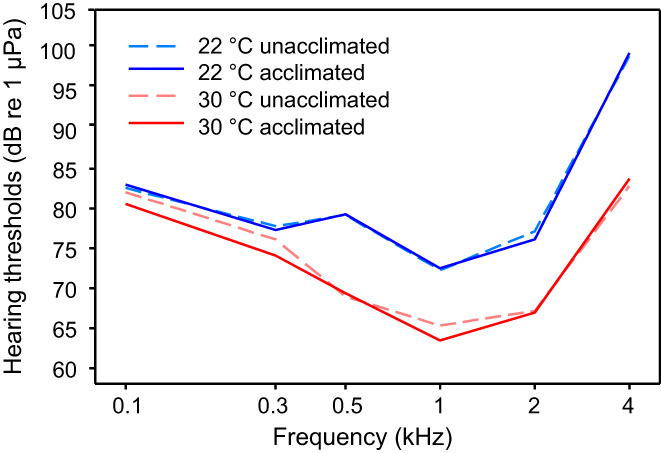
Mean auditory thresholds of unacclimated *Megalodoras uranoscopus* at 22 and 30°C (dashed lines) as compared to catfishes acclimated for 3–4 weeks to 22 and 30°C (solid lines). After Schliwa and Ladich (2021).

Increasing or decreasing temperatures raises the question if hearing sensitivities adapt to particular temperatures after some time of acclimation. Wysocki et al. (2009) reported acclimation effects in the eurythermal channel catfish when comparing unacclimated to acclimated animals after 4 weeks of acclimation. When the temperature was increased from 10 to 18°C or from 18 to 26°C (warm acclimation), hearing sensitivity increased in acclimated fish on average by 7 dB. No such effect, however, was found when temperatures were decreased (cold acclimation). Similar experiments in the doradid *M. uranoscopus* and the Pictus cat failed to confirm the previous result. Neither warm nor cold acclimation for 3 weeks changed hearing sensitivity in the latter two species (Figure 10) (Schliwa & Ladich, 2021).

No significant difference was observed in the minimum resolvable click periods of the auditory system between different temperatures in the Southern striped Raphael (Papes & Ladich, 2011). This indicates that this doradid catfish encodes the temporal information of sounds from conspecifics, independent of changes in ambient temperature.

### 4.2 Noise exposure

**FIGURE 11:**
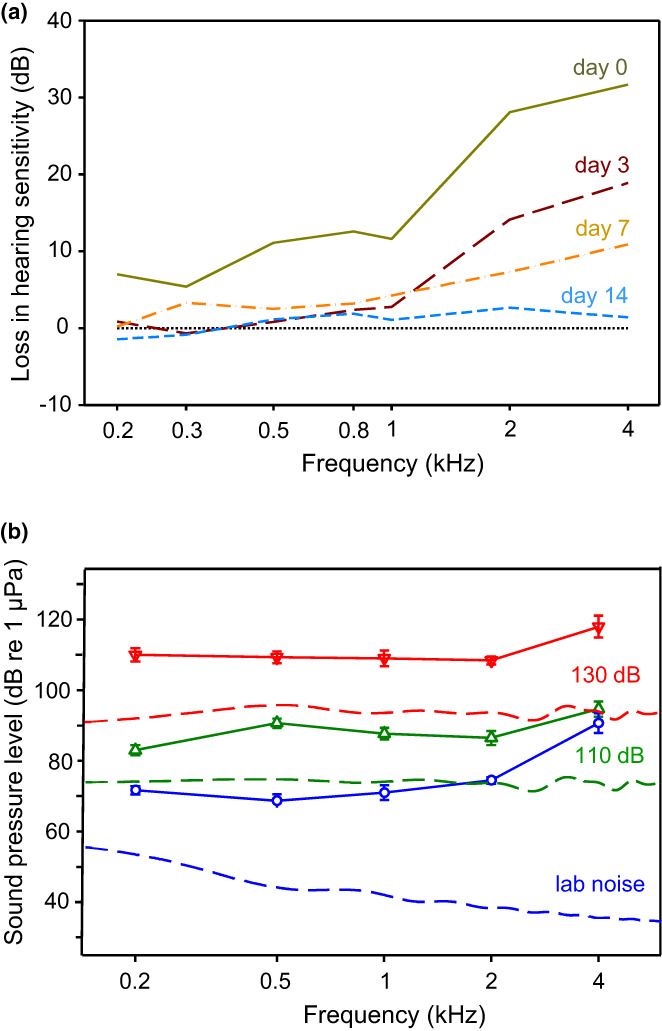
Effects of noise on hearing. (a) Mean temporary hearing loss in the Pictus cat after 12 h exposure to white noise (158 dB re 1 μPa). Dotted line at zero: baseline values. Day 0 shows the temporary threshold shift (TTS) immediately after noise exposure, days 3, 7 and 14 after 3, 7 and 14 days of recovery. Modified after Amoser and Ladich (2003). (b) Mean (± *SE*) hearing thresholds (solid lines) of the Southern striped Raphael and respective cepstrum‐smoothed noise spectra (dashed lines): Hearing thresholds obtained under normal laboratory conditions, masked hearing thresholds in the presence of white noise of 110 and of 130 dB. Modified after Wysocki and Ladich (2005).

Noise can negatively affect the ability of animals including fishes to orient or communicate acoustically (Brumm, 2013). Natural ambient or anthropogenic noise can affect hearing in several ways. Exposure to high‐level noise can result in a decrease in hearing sensitivity for several hours or days or even permanently after the noise has stopped. These phenomena are termed temporary threshold shift (TTS) or permanent threshold shift (PTS), respectively. Animals may recover from TTS after days or weeks to baseline levels. No recovery will be possible if auditory structures such as hair cells are irretrievably damaged (Yost, 1994). Exposing the Pictus cat to intense white noise (158 dB re 1 μPa) for 12 or 24 h resulted in a loss of auditory sensitivity up to 32 dB compared to baseline thresholds. Exposure duration of 12 or 24 h had no influence on hearing loss, TTS was highest immediately after exposure and in particular at highest frequencies (4 kHz) (Figure 11a). Hearing thresholds recovered within 14 days and returned to initial values (Amoser & Ladich, 2003).

Smith et al. (2006) and Smith and Monroe (2016) determined that sound‐induced hearing loss is correlated to saccular hair cell loss in goldfish and that hearing recovery is concomitant with hair cell regeneration. To address growing concern over the impact of anthropogenic sound on fishes, Halvorsen et al. (2013) exposed several species including the channel catfish to high‐intensity low‐frequency active naval sonar (LFAS). The stimuli consisted of frequency‐modulated sweeps ranging from 170 to 320 Hz lasting for 108 s and repeated three times. The maximum received sound pressure level (SPL) was 195 dB re 1 μPa. No effects on hearing were found in largemouth bass and yellow perch and only small effects (8–2 dB hearing loss between 200 and 1 kHz) in channel catfish. This indicates that damaging sound levels would be reached at a lower level in a species possessing accessory hearing structures such as Weberian ossicles in otophysines. Furthermore, mid‐frequency active sonar (MFAS) consisting of frequency sweeps from 2.8 to 3.8 kHz lasting for 2 s repeated five times at a sound exposure level of 220 dB re 1 μPa^2^s did not affect hearing sensitivity in rainbow trout and only in some catfish. Only one cohort of channel catfish showed a hearing loss of 4–6 dB at 2.3 kHz, but not at lower frequencies (Halvorsen et al., 2012). Thus, LFAS and MFAS had only minimal impact on a hearing specialist, the channel catfish, but none on species lacking auxiliary hearing structures.

### 4.3 Noise masking

Noise is an omnipresent environmental constraint on the auditory system of fishes and ultimately determines the detectability of sounds relevant to their orientation toward prey, predators and conspecifics, and to acoustic communication in their environment. Earlier studies by Tavolga (1967) and Buerkle (1969) showed that auditory thresholds are elevated in the presence of increased background noise in several marine teleosts, a phenomenon termed masking.

Hearing thresholds are typically measured in the presence of different noise types (white noise or natural noise recorded in the field) or different noise levels (Ladich, 2019). The main objectives are to investigate the extent to which white noise at naturally occurring sound pressure levels affects hearing thresholds. Playing back white noise at a masking noise level of 110 dB re 1 μPa increased hearing thresholds by up to 22 dB at 500 Hz in the vocal Southern striped Raphael (Figure 11b) (Wysocki & Ladich, 2005). The masking effect was much smaller at the highest frequency (4 dB at 4 kHz). At 130 dB, hearing thresholds increased by an additional 19 to 27 dB and were above 100 dB at all frequencies. To date, auditory sensitivity has not been measured in any catfish species in the presence of natural ambient noise. A study on the common carp (*Cyprinus carpio*, Cyprinidae) in which ambient noise of four different freshwater habitats (quiet backwater and lake, noisy stream and river) were played back showed that hearing thresholds were masked in the presence of all natural sound types (Amoser & Ladich, 2005). Catfish hearing is no doubt similarly masked. Thus, the detection of prey and predators and of conspecific sounds will be hindered in the presence of high natural noise levels.

## 5 DETECTION OF CONSPECIFIC SOUNDS

### 5.1 Neural representation of sounds within auditory pathways

Sounds produced by teleost fishes, including catfishes, typically constitute of series of pulses produced by various sonic organs (Ladich & Fine, 2006). Most sound‐generating mechanisms are based on rapidly contracting drumming muscles that variously (directly or indirectly) vibrate the swimbladder. Muscle contractions result in the emission of low‐frequency sound pulses and subsequently in the production of different sound types (Parmentier & Diogo, 2006). In contrast, pectoral mechanisms are based on rubbing of enhanced pectoral fin rays in the shoulder girdle such as in catfishes or on plucking of enhanced fin tendons, yielding series of high‐frequency sound pulses (Fine & Ladich, 2003; Ladich & Fine, 2006; Mohajer et al., 2015). Interestingly, representatives of some catfish families such as pimelodids and doradids possess two different sound‐producing organs. The result is the emission of swimbladder drumming and pectoral stridulation sounds, often simultaneously (Ladich & Maiditsch, 2020).

Temporal patterns of pulses within sounds are thought to be the most important carriers of acoustic information in vocal fishes. In order to investigate how conspecific sounds are processed by the auditory system, auditory evoked potentials (AEPs) elicited by conspecific sound pulses were investigated in catfishes. In the Southern striped Raphael (and the Pictus cat) each sound pulse elicited a separate brainwave that closely followed the temporal structure of sounds (Wysocki & Ladich, 2002, 2003).

**FIGURE 12:**
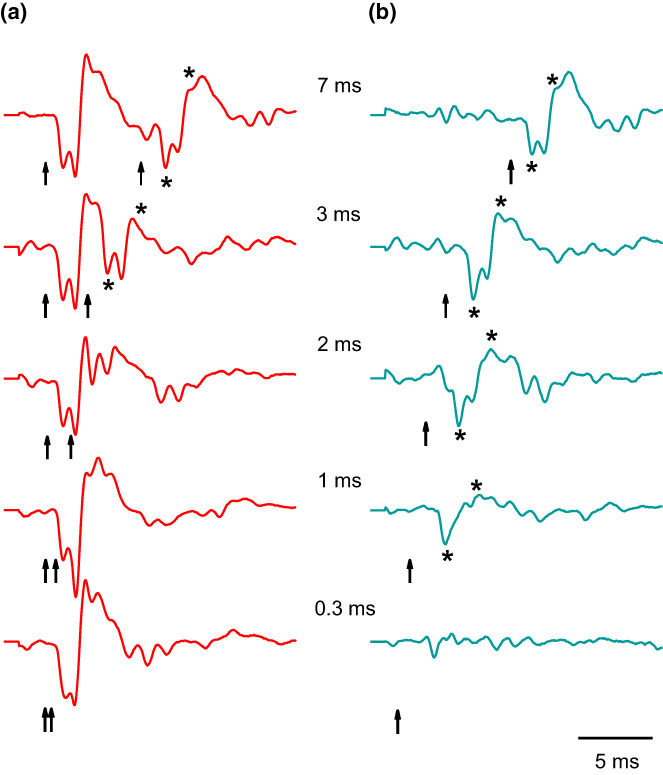
Temporal resolution of the auditory system. (a) AEPs of Southern striped Raphael in response to a double click stimulus (arrows) 32 dB above hearing threshold at different click periods (0.3–7 ms). (b) Response to the second click after a point‐to‐point subtraction of the AEP to a single click from that to the double click. Arrows: moments of click stimulation. Asterisks indicate the two reference peaks for analysis. Modified from Wysocki and Ladich (2002).

In order to determine whether fishes are able to utilize temporal characteristics of acoustic signals, time resolution was determined in otophysines including catfishes. This involved analyzing AEPs generated in response to double click stimuli with varying click periods. The response to the second click – after a point‐to‐point substraction of the AEP to a single click from that to the double click – indicates that pulse periods down to less than 1 ms are resolvable by the auditory system. The minimum pulse period resolvable by the auditory system at 32 dB above hearing level was 0.55 ms in the doradid catfish. Comparisons of the time resolution data of the auditory system to the pulse periods of intraspecific sounds revealed that Southern striped Raphael probably process each pulse within stridulation sounds (Figure 12) (Wysocki & Ladich, 2002).

**FIGURE 13:**
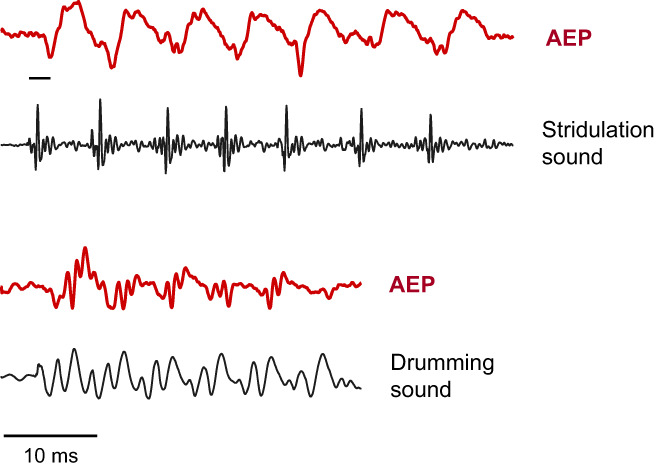
Auditory evoked potential (AEP) waveforms (bold lines) of the Pictus cat in response to a stridulation sound stimulus and a drumming sound stimulus (thin lines). The stridulation sound consists of seven consecutive pulses of variable pulse periods. The small horizontal bar at the left side indicates the latency (time) from the onset of a sound stimulus to the onset of the first AEP wave. Modified from Wysocki and Ladich (2003).

In order to investigate how conspecific sounds are processed by the auditory system, AEPs elicited by conspecific sounds were recorded in the Pictus cat. A previously recorded stridulation and a drumming sound were used as sound stimuli. The stridulation sound consisted of a series of seven consecutive pulses of variable amplitude and pulse periods (5.9–7.6 ms) (Figure 13). The stimulus pulse onsets and onsets of corresponding AEP waves were highly correlated. This indicates that the auditory system closely followed the temporal structure of communication sounds. Accordingly, temporal patterns, amplitude fluctuations and the frequency content of sounds can be represented in the auditory system and help the fish to extract important information for acoustic communication (Wysocki & Ladich, 2003).

### 5.2 Sex‐specific differences in sound detection

Sex‐specific differences in vocalizing behaviour and sound production have been observed in several taxa of teleosts (Ladich, 2015). Differences in auditory sensitivity were recorded in non‐related taxa, namely the African cichlid fish *Astatotilapia burton* (Cichlidae), the round goby (*Neogobius melanostomus*, Gobiidae) and the midshipman (*Porichthys notatus*, Batrachoididae) (Colleye et al., 2019; Maruska et al., 2012; Zeyl et al., 2013). This raises the question whether hearing sensitivity differs between sexes in any catfish species.

**FIGURE 14:**
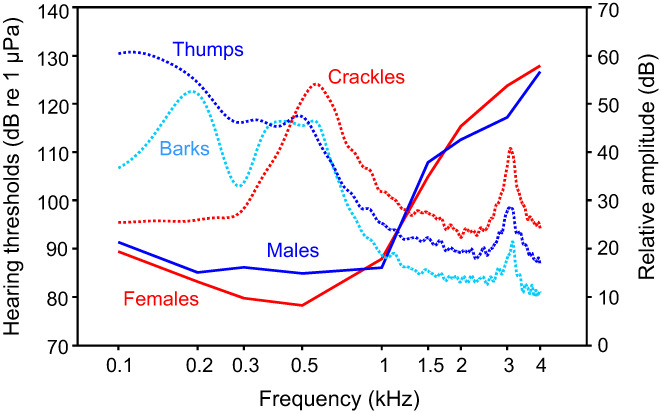
Mean hearing thresholds of male and female *Megalechis thoracata* (solid lines) and spectral characteristics of sounds (dotted lines). Power spectra of male barks and thumps and of female crackles are shown in relative amplitude values (right *Y*‐axis). Modified from Hadjiaghai and Ladich (2015).

Both sexes of the callichthyid armoured catfish *Megalechis thoracata* (Callichthyidae) detected frequencies between 100 Hz and 4 kHz. Females hear better between 0.1 and 1 kHz but not at higher frequencies (Figure 14) (Hadjiaghai & Ladich, 2015). Male and female *M*. *thoracata* differ in the size of their sonic organs and in the sounds produced. Pectoral spines of males are orange, in contrast to those of females, and are 1.7‐fold longer than in same‐sized females (Hadjiaghai & Ladich, 2015). Visual and acoustic threat displays differed between sexes. Males produced low‐frequency harmonic barks at longer distances and thumps at close distances, whereas females emitted broadband pulsed crackles when close to each other. Female sounds were shorter (167 ms vs. 219–240 ms) and of higher dominant frequency (562 Hz vs. 132–403 Hz) than those of males (Figure 14).

Both sexes showed best auditory sensitivities in frequency ranges where the main energies of sounds were concentrated (Figure 12). The greatest energy of sounds was concentrated from 180 to 620 Hz in males and from 470 to 750 Hz in female crackles. These data indicate that both sexes show a match between spectral content of sounds and best hearing ability below 1 kHz. The higher auditory sensitivity in females is partially based on hormone levels in teleost fish (Maruska et al., 2012) and most likely facilitates detection of male vocalization during nest advertisement, courtship and spawning.

## 6 MISCELLANEOUS

### 6.1 Functional significance of hearing in catfishes

Hearing will serve in detecting conspecific sounds in vocal fish species especially when the main energies of sounds are within the frequency range in which auditory sensitivities are highest. This is the case in pimelodid and doradid catfishes, which possess a large unencapsulated swimbladder and pronounced hearing at several kHz (Ladich, 1999; Lechner & Ladich, 2008). This does not hold true for species having tiny encapsulated bladders and limited hearing above 1 kHz. In the latter species, sound spectra and auditory sensitivities seem to be mismatched, for example, in the peppered corydoras (Ladich, 1999). Nevertheless, callichthyid catfish are quite vocal and produce courtship sounds before mating and aggressive sounds during territory defense (Hadjiaghai & Ladich, 2015; Pruzsinszky & Ladich, 1998).

### 6.2 Echolocation

A second function of the auditory system in catfishes was investigated by Tavolga (1971, 1976, 1977). The author hypothesized that short pulse‐type sounds constitute a coarse, short‐range echolocation system in the hardhead sea catfish. Observations of captive specimens demonstrated that they used low‐frequency sounds to detect and avoid obstacles. Muted animals bumped into the walls and became disorientated, whereas intact animals produced pulsed sounds and avoided barriers. Pulse‐type bursts have main energies below 300 Hz and match well with their best auditory sensitivities at 200 Hz (Figure 5a) (Popper & Tavolga, 1981). Accordingly, sound detection may also serve in catfish orientation besides acoustic communication with conspecifics.

### 6.3 Albinism

Pigmentation disorders such as albinism are occasionally associated with hearing impairments in mammals. Albinism is known in all classes of vertebrates and has been reported in numerous fish species including catfishes (Dingerkus et al., 1991). Lechner and Ladich (2011) studied AEP waveforms and hearing thresholds of normally pigmented and albinotic specimens of two catfish species, the European wels and the South American bronze corydoras (*Corydoras aeneus*, Callichthyidae). Neither auditory sensitivity nor the shape of AEP waveforms differed between the two types of specimens at any frequency tested in both species. That study indicated no association between albinism and hearing ability in catfishes and most likely other fish taxa. The authors hypothesized that hearing in fishes may be less affected by albinism than in mammals because of the lack of melanin in the fish's inner ear.

## 7 SUMMARY AND FUTURE RESEARCH

Catfishes have evolved the most elaborate and diverse structural adaptations for hearing and acoustic communication exhibited in any group of bony fishes. Two hundred years ago, Ernst Weber (1819, 1820) concluded based solely on dissections that catfish (and carps) possess a swimbladder which functions as an eardrum and hearing ossicles which transmit vibrations of the anterior bladder wall to the inner ear, similar to mammals. It took 100 years until Von Frisch (1923) proved experimentally – by conditioning a brown bullhead to respond to whistles when being fed – that Weber was right. Catfish and minnows hear excellently compared to other fish groups lacking accessory hearing structures. Experimental elimination of the swimbladder and (Weberian) ossicles decreased hearing abilities, which are otherwise similar to those of humans.

Adapting an electrophysiological technique to fish (auditory evoked potential AEP technique), which is otherwise used by physicians to measure hearing in newborns, proved to be helpful to determine various hearing abilities in catfish without conditioning. The AEP technique has increased the number of catfish species whose baseline hearing thresholds (audiograms) were determined from 2 to 28 species (Ladich & Fay, 2013). It furthermore showed that hearing improvement during ontogeny depends on development of the chain of ossicles. Species with large swimbladders and 3–4 ossicles hear better at higher frequencies (1–6 kHz) than species having tiny bladders and 1–2 ossicles.

Furthermore, ecological factors such as temperature and ambient noise affect hearing abilities. While catfish hearing improves with temperature, noise decreases auditory sensitivity, exposure to high‐level noise (>150 dB) resulted in a temporary threshold shift (TTS) and recovery of hearing after several days. Low noise levels result in a decline due to masking but no TTS.

Catfish generate pulsed sounds either by rapidly contracting swimbladder muscles (drumming sounds) or by rubbing the pectoral spines in a groove of the shoulder girdle (stridulation sounds). AEPs elicited by conspecific sounds in the Pictus cat indicate that pulse periods down to less than 1 ms are resolvable by the auditory system and thus detectable by conspecifics.

This basic knowledge about hearing raises numerous questions deserving future investigation. A first task would be to clarify which constraints are responsible for the selection of the diversity of peripheral auditory structures, namely swimbladders and Weberian ossicles. Why is such a diversity lacking in closely related cypriniforms? The “ecological constraints hypothesis” states that hearing sensitivity evolved in parallel to ambient noise levels (Ladich, 2014). To test that hypothesis, auditory sensitivities need to be measured under natural noise conditions. At natural noise levels, the catfishes' hearing should not be masked.

The ontogenetic development of the Weberian apparatus and auditory sensitivity needs to be investigated in both types of catfishes – those with large swimbladders and a full set of hearing ossicles and those with two tiny encapsulated bladders and a reduced number of ossicles. That approach will clarify when both groups start to differ in swimbladder and ossicle anatomy and auditory sensitivity.

Furthermore, we need to know if the diversity in swimbladder anatomy and auditory ossicles in catfishes is paralleled by a diversity in the inner ear anatomy and fine structure. Knowledge on sensory epithelia (maculae) in catfish inner ears is limited (Jenkins, 1977, 1979, Popper & Tavolga, 1981, reviewed in Ladich & Bass, 2003). A comparative investigation of sensory epithelia (maculae) in cichlid fish in parallel to the diversity of their swimbladders showed an enlargement of all three maculae in the orange chromide (*Pseudetroplus maculatus*, Cichlidae), a species which possesses the largest swimbladder that is directly connected to the inner ears (Schulz‐Mirbach et al., 2014).

In several catfish families, swimbladders serve (in addition to buoyancy) in sound production (via rapid contraction of drumming muscles) and in hearing. This raises the question if the inner ear might not be overpowered during large‐amplitude swimbladder vibrations. Interestingly, pimelodid catfish possess a small second swimbladder muscle (tensor tripodis muscle) which inserts rostrally close to the tripus, the most caudal Weberian ossicle (Ladich, 2001; Ladich & Bass, 2003; Ladich & Fine, 1994). Bridge and Haddon (1892) and Schachner (1977) suggested, based on anatomical grounds, that its contraction would attenuate or eliminate tripus vibrations and thus sound conduction to the inner ear. Physiological experiments are needed to clarify whether an ear protection mechanism exists in catfishes, analogous to the tympanal reflex in mammals. In mammals, contractions of the tensor tympani muscle increases tension on the tympanum and limits motion of ossicles.

Most importantly, future research should focus on aquatic noise to clarify if and to what degree anthropogenic noise affects hearing, either via masking or TTS, and how this affects sound communication and subsequently reproduction in catfishes in natural environments.

## Data Availability

Data sharing not applicable to this article as no datasets were generated or analysed during the current study.
